# Suppressing Blood-Cell Migration Lag via Dean-Cycle Phase Regulation Enables High-Purity CTC Enrichment in an Inertial Microfluidic Array

**DOI:** 10.3390/mi17040446

**Published:** 2026-04-03

**Authors:** Taihang Wu, Haozheng Li, Xiange Sun, Xiaodong Ren, Hong Wang, Qing Huang

**Affiliations:** 1 Department of Laboratory Medicine, Daping Hospital, Army Medical University, Chongqing 400042, China; 18234890524@163.com (T.W.);; 2 Department of Biomedical Materials Science, Army Medical University, Chongqing 400038, China

**Keywords:** spiral inertial focusing, arrayed chip, circulating tumor cell, label-free, dean vortices

## Abstract

Circulating tumor cells (CTCs) are valuable liquid-biopsy biomarkers, yet their extreme rarity makes high-purity, high-throughput enrichment challenging. In spiral inertial microfluidics, high cell loading induces long-range hydrodynamic interactions that broaden the focused blood-cell stream; consequently, a subpopulation completes the ~0.5 and ~1.0 Dean-cycle migrations with a phase delay, compressing the CTC–blood cell gap and degrading purity. Here we propose a Dean-cycle phase-regulated double-spiral design informed by this phenomenon. This design aims to mitigate the stream-broadening effect by boosting the Dean number during the first half-cycle to promote synchronized blood-cell migration and shifting the CTC equilibrium position near one full cycle to further widen the CTC–blood cell separation. We implement this strategy in a second-generation double-spiral microfluidic chip (SDMC) and scale it to a four-channel parallel array (ASDMC). Under optimized conditions, ASDMC processes diluted whole blood (hematocrit = 4%) without the need for red blood cell (RBC) lysis or antibody labeling, achieving a sample throughput of 1200 μL·min^−1^. Specifically, it exhibits a mean recovery rate of 98.8% across three spiked tumor cell lines (MCF-7, PC-9, and Mahlavu) and a mean white blood cell (WBC) depletion efficiency of 93.3%. In a pilot clinical testing of 20 patients (NSCLC and HCC), enriched fractions enabled immunofluorescence identification of CK^+^CD45^−^DAPI^+^ CTCs, with an exploratory trend of increasing CTC counts with advanced disease stage (4–34 cells·mL^−1^). These results describe a scalable, label-free platform, and the observed purification performance aligns with our proposed mechanism: Dean-cycle phase regulation to mitigate blood-cell migration lag. Our findings support further technical validation and clinical assessment in larger cohorts.

## 1. Introduction

Circulating tumor cells (CTCs) are rare tumor-derived cells that detach from primary or metastatic lesions and enter the peripheral bloodstream [1]. As clinically valuable “liquid biopsy” biomarkers, CTCs enable minimally invasive early cancer detection and prognosis assessment, support therapeutic monitoring [2], and provide insights into tumor heterogeneity [3] and drug-resistance mechanisms [4,5]. However, their extreme rarity—often only a few cells per milliliter among 10^7^–10^9^ blood cells [6,7,8]—makes rapid and high-purity enrichment challenging [9].

Current CTC enrichment technologies are generally classified into biological property-based and physical property-based methods [10]. Immunoaffinity-based techniques (e.g., EpCAM/cytokeratin targeting) offer high specificity [11,12,13,14,15,16]; however, they are vulnerable to heterogeneous or downregulated marker expression [10] and typically involve costly reagents [17] and multi-step workflows [18] that may compromise cell viability and limit downstream analyses [19,20]. In contrast, physical property-based technologies exploit intrinsic biophysical differences (e.g., cell size, density, deformability, electrical properties, or acoustic response) to enable label-free processing [10]. Representative examples include the Isolation by Size of Epithelial Tumor Cells (ISET) filtration system [21], density-gradient centrifugation [22], dielectrophoretic separation [23], acoustic standing-wave sorting [24], and, most notably, inertial microfluidics. Nevertheless, many conventional label-free implementations suffer from membrane clogging [25], cell loss [26], or limited throughput [27], which can compromise separation efficiency and clinical scalability.

Against this background, inertial microfluidics provides an attractive alternative due to its passive operation, high throughput, and scalability. This technique harnesses the interplay between size-dependent inertial lift and curvature-induced Dean drag forces to enable cell self-focusing and continuous separation [28]. Over the past decade, diverse microchannel geometries have been optimized to improve hydrodynamic precision and separation resolution [29,30,31,32,33,34,35,36,37,38]. Numerous studies have shown that fine-tuning channel geometry, branch flow resistance, and secondary flow regulation can effectively alter particle and cell migration trajectories, enhancing downstream separation performance [29,30,31]. For example, spiral [29,32] and slanted-spiral [33,34] channels utilize secondary flows focusing under moderate-to-high Reynolds numbers; labyrinth [35] or serpentine [36] channels enhance Dean vortices via sharp turns; micro-post array [37] channels employ deterministic lateral displacement for fine trajectory control; and hydrofoil-structured channels have been developed to modulate secondary flow fields, improving CTC capture efficiency [38]. Moreover, hybrid systems integrating spiral inertial units with viscoelastic or magnetophoretic modules [39] extend applicability to large-volume samples while preserving cell viability.

Beyond local inertial focusing, the overall performance of a multiplexed microfluidic chip also depends on global flow-network characteristics, such as hydraulic resistance, branch-to-branch flow balance, pressure drop, wall shear stress, and manifold geometry [40]. For pressure-driven microfluidic systems, these factors critically determine whether parallel units operate under consistent hydrodynamic conditions, which in turn affects separation stability, throughput scalability, and cell integrity [41]. Prior work has demonstrated that minimizing and balancing hydraulic resistance is essential for achieving uniform flow distribution in high-flow-rate, small-footprint microfluidic architectures [41], while the electric circuit analogy provides an efficient tool for predicting pressure/flow relationships in complex channel networks [42]. Specifically, systematic studies on bypass channel design have shown that adaptive resistance tuning through branch channel geometry optimization can effectively suppress upstream pressure feedback and stabilize flow partitioning in cascaded microfluidic systems [30,43]. This provides the core engineering rationale for the balancing channel design in this work. Importantly, such resistance-based design principles have also supported advanced biomedical microfluidic applications, including cell analysis and immunotherapy-related platforms [44]. These considerations underscore that the design of high-performance CTC chips requires not only optimization of channel-scale focusing physics, but also careful regulation of chip-level flow resistance and parallel-network architecture [32,45].

Our group previously developed the first-generation double-spiral microfluidic chip (FDMC) for CTC enrichment [46] and successfully applied it to gene mutation analysis [47]. Nevertheless, its throughput and purification performance were insufficient for routine clinical use, and the device footprint limited system integration.

Here, we present an arrayed second-generation double-spiral microfluidic chip (ASDMC) that integrates four optimized second-generation units (SDMCs) in parallel. Motivated by our observation that long-range hydrodynamic interactions broaden the focused blood-cell stream under high blood-cell loading in spiral inertial microfluidics [48,49], we hypothesize that this stream broadening arises from a phase delay in blood-cell migration—defined here as the “lag effect”, where a subpopulation of blood cells completes the ~0.5 and ~1.0 Dean-cycle migrations asynchronously. Based on this hypothesis, we introduce a Dean-cycle phase-regulated strategy with section-wise geometry programming. This strategy is designed to promote blood-cell migration synchrony during the first half-cycle and shift the CTC focusing position near one full cycle, with the aim of widening the CTC–blood cell lateral separation. Guided by numerical optimization (MATLAB R2021b and COMSOL Multiphysics 6.3), we optimized the geometric and flow-resistance balance, which helped to determine the optimal dimensions before refining the curvature design. Finally, we characterize the analytical performance of the proposed platform through spike-in experiments in healthy donor blood using multiple tumor cell lines, and further assess its preliminary clinical application potential by enriching and enumerating CTCs from a small pilot cohort of clinical patient samples.

## 2. Materials and Methods

### 2.1. Modeling Method

To enable footprint reduction for array integration while preserving CTC enrichment performance, we re-designed the key structural parameters governing particle focusing and separation, namely the channel geometry and curvature ratio [50]. Meanwhile, SDMC was built on the two-stage cascaded-spiral architecture of FDMC [46], with each spiral segment comprising 1.75 turns of an Archimedean spiral. According to Equations (S3) and (S12) in Supplementary Methods S2, particle migration is primarily governed by the channel cross-sectional dimensions and the radius of curvature. Therefore, MATLAB R2021b (The MathWorks, Natick, MA, USA) was employed for theoretical modeling to optimize the key structural parameters of the two-stage spiral channels.

#### 2.1.1. Cross-Section Selection Guided by the Force Balance Ratio $\left(R_{f}\right)$ and the Confinement Ratio

**Figure 1:**
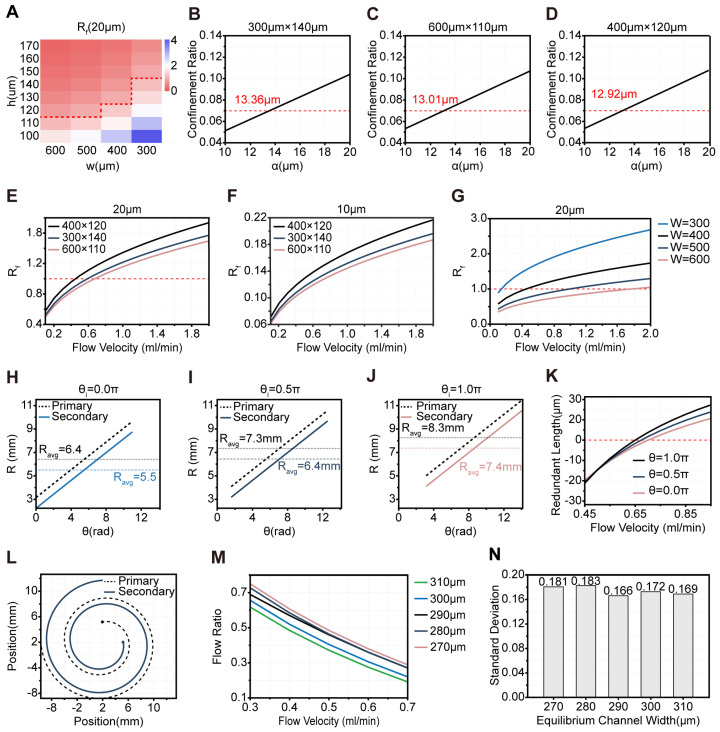
Parameter Optimization of SDMC. (**A**) Simulation of the $R_{f}$ for 20 µm particles at a flow rate of 800 μL·min^−1^ under different channel cross-sectional dimensions. The red dashed line separates the channel cross-sectional dimensions with $R_{f}>1$ and $R_{f}<1$ under this condition. (**B**–**D**) Confinement ratio analysis for particles in the top three cross-sectional geometries with the largest minimum focusable size thresholds. The red dashed line indicates a threshold value of 0.07. (**E**,**F**) Dependence of the $R_{f}$ of 20 μm and 10 μm particles on flow rate for the three screened cross-sectional geometries. (**G**) Variation of $R_{f}$ with flow rate for multiple channel heights (h) at fixed width (w) for 20 μm particles. (**H**–**J**) Changes in the mean curvature radius $R_{\mathrm{avg}}$ of the primary and secondary spirals at initial angular positions $\left(\theta_{i}\right)$ of 0.0π, 0.5π, and 1.0π as a function of angular position. (**K**) Redundant length at different $\theta_{i}$, defined as the difference between the spiral arc length for 1.75 turns $\left(L_{S}\right)$ and the migration length required for a 10 μm particle to complete one Dean cycle at the same $\theta_{i}$ $\left(L_{D}\right)$, as a function of flow rate. (**L**) Schematic of the selected configurations for primary and secondary spirals. (**M**) Evaluation of flow ratio variation at different balancing channel widths. (**N**) Bar chart showing the standard deviation of the mean flow-ratio values in (**M**).

To provide the mean curvature radius input required by Equations (S3)–(S5) in Supplementary Methods S2, we first parameterized the spiral centerline in MATLAB and used $a_{2}=2.3\mathrm{mm}$, d=3.7 mm, and an initial angular position $\left(\theta_{i}=\pi \right)$ as the baseline geometry for subsequent calculations. According to Equations (S9)–(S12) in Supplementary Methods S2, we computed $R_{f}$ for 20 µm and 10 µm particles under different channel width–height combinations at a reference flow rate of 800 μL·min^−1^ (Figure 1A and Figure S1A). For preliminary channel design, we adopt both $\alpha_{p}/D_{h}>0.07$ and $R_{f}\geq 1$, where $R_{f}$ is the ratio of inertial lift force to Dean drag force. This criterion is widely used in inertial microfluidics design to identify conditions where inertial lift dominates over Dean drag, enabling stable particle focusing [50,51]. Geometries with $R_{f}<1$ were first excluded to ensure 20 µm focusing; then the minimum focusable size was back-calculated from $\alpha_{p}/D_{h}=0.07$ for each candidate. Since WBCs are typically 10–15 μm in size, whereas CTCs are generally larger than 15 μm [51], a larger minimum focusable size theoretically leads to less focusing of WBCs. Following this principle, we selected the top three channel geometries with the largest minimum focusable size thresholds: 300 μm × 140 μm (13.36 μm), 600 μm × 110 μm (13.01 μm), and 400 μm × 120 μm (12.92 μm) (Figure 1B–D). Although the 400 μm × 120 μm channel had a slightly lower minimum focusable size threshold than the other selected geometries, it showed superior $R_{f}$ for 20 μm particles under all tested flow rates (Figure 1E). Furthermore, for 10 μm particles, the $R_{f}$ in this cross-section is consistently much less than 1 over the entire flow rate range (Figure 1F). As reported in the literature, curved channels enable the differential separation of particles according to their $R_{f}$ values [28]. Thus, at a given flow rate, a higher $R_{f}$ for the 20 μm particles within a certain range corresponds to a larger separation distance between these particles and those of other sizes. Based on this observation, we selected the 400 × 120 μm cross-section as the initial one. To examine how sensitive the initial cross-section screening at 800 μL·min^−1^ is to deviations in flow rate, we evaluated the dependence of $R_{f}$ for the 20 μm particles on flow rate for different channel dimensions. When the channel height was fixed at 120 µm, a channel width of 400 µm allowed $R_{f}$ to exceed 1 at relatively low flow rates while achieving the highest throughput (Figure 1G). Similarly, when the channel width was fixed at 400 µm, the same optimal performance was observed (Figure S1B). These comparisons identified 400 µm × 120 µm as the most robust cross-section, which was therefore used in all subsequent simulations and experiments.

#### 2.1.2. Spiral Channel Design and the Relationship Between Initial Angular Position $\left(\theta_{i}\right)$ and Mean Curvature Radius $\left(R_{\mathrm{avg}}\right)$

Given a fixed number of turns, $R_{\mathrm{avg}}$ of both the primary and secondary spirals is directly proportional to their initial radius (a) and initial angular position $\left(\theta_{i}\right)$. According to Equation (S12) in Supplementary Methods S2, $R_{f}$ is also proportional to $R_{\mathrm{avg}}$, meaning that $R_{f}$ increases with larger values of a and $\theta_{i}$. The values for a have been specified in the Supplementary Methods S1. For system coordination and subsequent process tuning, $\theta_{i}$ of 0π, 0.5π, and 1π were considered (Figure S1C–E).

In the spiral microchannel design, the primary spiral is mainly responsible for stable focusing of CTCs, while the secondary spiral plays a critical role in the size-based separation of different cell types. Due to the intrinsic structure of the chip—the primary spiral located on the outside and the secondary on the inside—the initial radius of the secondary spiral $\left(a_{2}\right)$ is inevitably smaller than that of the primary spiral $\left(a_{1}\right)$, resulting in a smaller $R_{\mathrm{avg}}$ for the secondary spiral (Figure 1H–J). This reduces the $R_{f}$ in the secondary spiral, causing the focusing position of CTCs to move away from the inner wall, thereby decreasing the recovery of CTCs.

However, both theoretical analysis and simulations demonstrate that increasing the $\theta_{i}$ of the spiral channel significantly enhances its $R_{\mathrm{avg}}$ (Figure 1H–J), thereby increasing the $R_{f}$ for CTCs and improving their focusing performance. Therefore, to ensure the focusing stability within the secondary spiral, its initial angular position should be set greater than that of the primary spiral. Notably, the arc length for 1.75 turns $\left(L_{S}\right)$ varies at different $\theta_{i}$ (Figure S1F), which further illustrates the effect of $\theta_{i}$ on the geometry and flow characteristics within the channel. Simulations were performed for 20 μm and 10 μm particles in the secondary spiral under various $\theta_{i}$, revealing that the migration length required for 20 μm particles to achieve focusing $\left(L_{I}\right)$ is shorter than that required for a 10 μm particle to complete one Dean cycle (DC) $\left(L_{D}\right)$ (Figure S1F); thus, the channel length was ultimately determined by the latter. According to Equations (S13)–(S15) in Supplementary Methods S2, $L_{I}$ is independent of the $R_{\mathrm{avg}}$ of the channel, and thus $L_{I}$ remains constant under different $\theta_{i}$. In contrast, for 10 μm particles, $L_{D}$ is related to the $R_{\mathrm{avg}}$, resulting in variations in $L_{D}$ at different $\theta_{i}$. It should be explicitly stated that this conclusion is derived from a first-order approximation based on cross-section-averaged flow parameters. In practical scenarios, both $L_{I}$ and $L_{D}$ are subject to the influences of spatially varying curvature along the spiral channel, local Reynolds number fluctuations, and channel cross-sectional geometry. This simplified correlation is solely employed for the preliminary geometric screening of the spiral architecture, and does not represent a universal physical law for all curved microfluidic systems.

Further simulation results show that the redundant length—defined as the difference between the $L_{S}$ and the corresponding $L_{D}$ value at the same $\theta_{i}$—is minimized when the $\theta_{i}$ is set to 0π or 0.5π (among the three tested $\theta_{i}$), which benefits downstream fine-tuning (Figure 1K). Based on these findings, the $\theta_{i}$ was finally set to 0π for the primary spiral and 0.5π for the secondary spiral (Figure 1L). Simulations confirm that, under these configurations, both spirals yield $R_{\mathrm{avg}}$ of approximately 6.4 mm. This indicates that the focusing stability of CTCs in the secondary spiral is comparable to that in the primary spiral, thereby accomplishing the desired optimization.

### 2.2. Inter-Stage Hydrodynamic Decoupling and Balancing Channel Width

In our setup, the primary circulating CTC outlet is directly connected to the sheath 2 channel (Figure S2A). As a result, fluctuations in the sheath 2 flow can propagate upstream, potentially affecting the flow dynamics in the primary spiral channel. To mitigate pressure feedback between the spiral channels, we redesigned the waste 1 channel to function as a balancing channel, providing adaptive resistance. We performed systematic fluid dynamics simulations to evaluate the system’s hydrodynamic stability under different balancing channel widths. The standard deviation of the flow rate ratio between the primary CTC outlet and the waste 1 outlet, across varying sheath 2 flow conditions, was used as the core metric for evaluating decoupling performance. Detailed simulation settings are provided in Supplementary Methods S4 and Table S1. The simulation results showed that the optimal balancing channel width ranges from 270 to 310 µm (Figure 1M). The minimum standard deviation of the flow rate ratio (0.166) was achieved at a width of 290 µm (Figure 1N), corresponding to the optimal pressure buffering effect and stable flow partitioning under our tested operating conditions; a representative simulation result under this optimal width is shown in Figure S3A.

### 2.3. Design Principle

**Figure 2:**
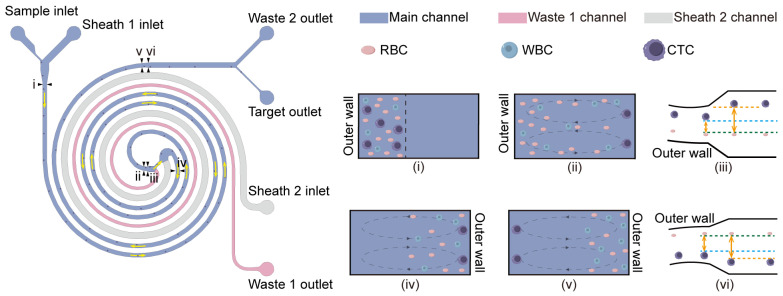
Schematic of CTC Separation Principle in Phase-Dependent SDMC. Flow patterns and cell trajectories in the SDMC. (**i**,**ii**) Cell positions at the inlet and outlet of the primary spiral. (**iv**,**v**) Cell positions at the inlet and outlet of the secondary spiral. (**iii**,**vi**) Schematic diagrams illustrating cell migration driven by aspect-ratio changes at the end of the primary and secondary spirals, where blue and orange dashed lines indicate CTC trajectories, the green dashed line indicates the blood cell trajectory, and the orange two-way arrow denotes the lateral distance between CTCs and blood cells. The yellow unidirectional arrow represents the movement direction of CTCs within the chip channel.

The primary spiral serves as a coarse separation stage by removing most red blood cells (RBCs) and part of the white blood cells (WBCs) (Figure S4A), whereas the secondary spiral functions as the purification stage to further separate CTCs from residual blood cells (Figure S4B). The two spiral stages are arranged in parallel with opposite flow directions, ensuring that cells enter from the outer wall in each spiral (Figure 2 (i,iv)). Within each spiral stage, blood cells migrate from the outer wall to the inner wall and back to the outer wall, completing ~1.0 DC, whereas CTCs progressively focus near the inner wall (Figure 2 (ii,v)). This positional difference is exploited to separate CTCs from blood cells by increasing their lateral separation, defined as the lateral distance between the CTC position and the blood-cell position when blood cells complete ~1.0 DC.

However, under high blood-cell loading, long-range hydrodynamic interactions broaden the focused blood-cell stream, generating a phase-lag subpopulation that fails to complete the expected ~0.5 DC and ~1.0 DC migrations within the prescribed channel length. As a result, residual blood cells encroach on the CTC stream near the inner wall, narrowing the lateral separation and increasing carryover into the target outlet, thereby reducing purity. To suppress this effect, we apply a Dean-cycle phase–regulated strategy in the purification stage: (i) during the first half-cycle (~0.5 DC), we increase the Dean number (De) to promote the synchrony of blood cell migration toward the inner wall; and (ii) near one full cycle (~1.0 DC), we shift the CTC focusing position toward the inner wall to further widen the CTC–blood cell separation.

Building on this concept, the purification stage was subdivided into three functional sections (Figure S4B), each designed with distinct geometric parameters to regulate particle behavior around 0.5 DC and 1.0 DC. Section I serves as the transition region between the primary and secondary spirals. According to Equations (S3)–(S5) in Supplementary Methods S2, De is negatively correlated with the $R_{\mathrm{avg}}$. Therefore, by reducing the $R_{\mathrm{avg}}$ in Section I $\left(R_{\mathrm{avg}}=0.8\;\mathrm{mm}\right)$, De can be significantly increased, thereby strengthening Dean-driven secondary flow and enhancing the synchronous migration of blood cells during the first 0.5 DC, while effectively reducing the lag effect (Figure S4B(iii)).

Section II corresponds to the secondary spiral and serves as the main purification segment. In this region, CTCs progressively focus and eventually stabilize near the inner wall. In the initial portion of Section II, the strong Dean-driven secondary flow (enabled by the smaller curvature radius) further promotes synchronized completion of the first ~0.5 DC by blood cells, thereby suppressing the lag effect. As blood cells approach the completion of ~1.0 DC within the latter portion of Section II, a relatively clear lateral separation from the inner-wall-focused CTC stream is established. However, because the curvature radius increases gradually along the downstream part of Section II, the secondary flow diminishes in the second half-cycle, and residual phase lag of blood cells becomes difficult to fully eliminate. Therefore, a terminal straight Section III is introduced to further enlarge the CTC–blood cell gap near ~1.0 DC, improving final purification at the target outlet.

Section III is the terminal straight channel of the purification stage. Here, the channel width is expanded from 400 μm in Section II to 500 μm in Section III, thereby increasing the aspect ratio (w/h), which drives the equilibrium focusing position of CTCs further toward the inner wall (Figure 2 (vi)). Previous studies and classical theories have demonstrated that an increased channel aspect ratio shifts the particle equilibrium position toward the inner wall [28]. In contrast, blood cells experience only a very small inertial lift force $\left(F_{L}\right)$ in the straight channel; as a result, their lateral positions are barely affected by changes in aspect ratio (Figure 2 (vi)). This design further enlarges the lateral gap between CTCs and blood cells after blood cells have completed approximately 1.0 DC, thereby enhancing the final separation efficiency. Moreover, we also applied this design at the end of the primary spiral to ensure that the preliminarily focused CTCs could be stably guided into the secondary spiral channel (Figure 2 (iii)).

To further improve the device’s throughput while maintaining optimized flow control, four SDMCs are integrated coplanarly to form an ASDMC (Figure S5A,B). This arrangement ensures uniform flow conditions across the parallel channels and maintains a separation efficiency comparable to that of a single SDMC after array integration. The detailed fabrication methods are described in Supplementary Methods S5.

### 2.4. Statistical Analysis

All data are presented as mean ± standard deviation (SD). The definition of replicates for each experiment is provided in the corresponding figure legends. Unless otherwise specified, all experiments were performed with at least three independent replicates, defined here as independent chip-sorting experiments using different freshly prepared samples. Repeated measurements within the same experiment were used only to confirm measurement consistency and were not treated as independent replicates for statistical analysis of device performance.

Before parametric analyses, data normality was assessed using the Shapiro–Wilk test, and homogeneity of variance was evaluated using Levene’s test. Comparisons between two independent groups were performed using a two-tailed unpaired Student’s *t*-test, whereas paired comparisons (e.g., cell viability before and after sorting) were analyzed using a two-tailed paired Student’s *t*-test. Comparisons among three or more groups were performed using one-way analysis of variance (ANOVA), followed by Tukey’s post hoc multiple pairwise comparisons. A *p* value < 0.05 was considered statistically significant. Statistical significance is indicated as follows: * *p* < 0.05, ** *p* < 0.01, and *** *p* < 0.001. All statistical analyses were performed using Origin version 2024 (OriginLab, Northampton, MA, USA). The FDMC design was adopted from a previously published study [46]; however, all FDMC-related experimental data reported here were generated independently in this work.

## 3. Results

### 3.1. Systematic Optimization of Sorting Conditions for the SDMC

According to Equations (S3), (S6), (S11) and (S15) in Supplementary Methods S2, the migration lengths required for RBCs and WBCs to complete one Dean cycle in spiral microfluidic chips are theoretically identical under the rigid spherical particle model [28,51,52]. This theoretical conclusion forms the basis of our preliminary channel design, and we explicitly note its simplifying nature: in reality, RBCs and WBCs have intrinsic differences in size, deformability, and hydrodynamic behavior, which may lead to deviations in their actual migration trajectories in the spiral channel. The validity of this design framework is ultimately verified through whole-blood sorting experiments in subsequent sections. However, the concentration of RBCs in blood (4–6 × 10^9^ cells·mL^−1^) is much higher than that of WBCs (4–10 × 10^6^ cells·mL^−1^) [8]. To ensure high separation purity, 6 μm and 20 μm microspheres were used in this study to respectively simulate RBCs and CTCs for optimization of separation conditions. The corresponding particle concentrations and detailed usage protocols are described in Supplementary Methods S6. The sample inlet flow rate (300 μL·min^−1^) was selected based on previously reported optimal parameters for FDMC [46]. According to Equation (S6) in Supplementary Methods S2, $Re_{c}$ for the primary and secondary spiral channels were calculated using the respective total flow rates: the sum of the sample flow and sheath 1 flow for the primary spiral, and the sum of the primary CTC outlet flow and sheath 2 flow for the secondary spiral.

#### 3.1.1. Optimization of Flow Rates for Primary and Secondary Spirals

**Figure 3:**
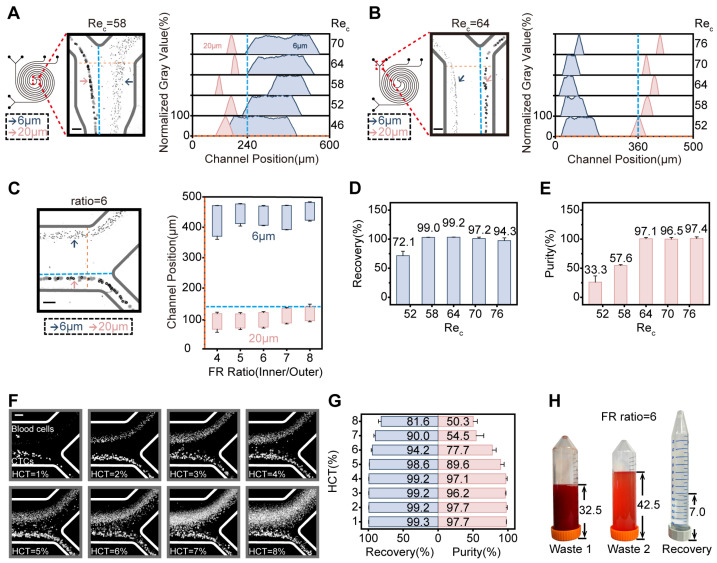
Fluid Parameter Regulation and CTC Enrichment Performance Verification of SDMC. (**A**,**B**) Effect of flow rates on particle focusing behavior in the primary (**A**) and secondary (**B**) spiral channels. The channel Reynolds numbers $\left(Re_{c}\right)$ shown in the figure was calculated from the corresponding flow rates. Scale bar: 100 μm (**C**) Effect of flow resistance ratio (FR ratio, inner/outer) on particle focusing behavior in the secondary spiral channel. Scale bar: 100 μm; *n* = 3 independent biological replicates for each tested FR ratio condition. (**D**,**E**) Recovery and purity of Mahlavu cells at different sheath 2 flow rates under the optimal sheath 1 flow rate; *n* = 3 independent biological replicates for each tested flow rate condition. (**F**,**G**) Schematic illustration of cell separation at the outlet of the secondary spiral channel (**F**) and the corresponding recovery and purity of Mahlavu cells (**G**) at different HCT levels (1–8%) under optimized flow rates and flow resistance. Scale bar: 100 μm; *n* = 3 independent biological replicates for each tested HCT level. (**H**) Comparison of collected volumes (in mL) from the three SDMC outlets; *n* = 3 independent biological replicates.

Within the SDMC, the sample flow rate was kept constant while the sheath 1 flow rate was systematically varied. The optimal operating conditions for the primary spiral channel were identified by analyzing the focusing and migration trajectories of 20 μm and 6 μm microspheres. In this channel, effective separation of 20 μm and 6 μm particles was achieved within the $Re_{c}$ range of 46–70 (Figure S6A), with the best separation observed at $Re_{c}=58$ (Figure 3A), corresponding to an optimal sheath 1 flow rate of 600 μL·min^−1^. Under this optimal condition, the flow rate at the primary CTC outlet was determined to be 200 μL·min^−1^, with detailed methodologies described in Supplementary Methods S7. On this basis, the sheath 2 flow was adjusted to optimize focusing and sorting in the secondary spiral channel. For the secondary spiral, effective separation was achieved within the $Re_{c}$ range of 52–76 (Figure S6B), with the best separation occurring at $Re_{c}=64$ (Figure 3B), corresponding to a sheath 2 flow rate of 700 μL·min^−1^.

#### 3.1.2. Optimization of Flow Resistance Ratio for Three SDMC Outlets

Furthermore, the flow resistance balance among the three SDMC outlets influences particle focusing. By designing the width of waste 1 channel appropriately, its impact on the primary spiral flow was minimized; thus, analysis focused on the flow resistance ratio (FR ratio) between the target outlet (inner) and waste 2 outlet (outer). Under the previously determined optimal flow rates of 300 μL·min^−1^ (sample), 600 μL·min^−1^ (sheath 1), and 700 μL·min^−1^ (sheath 2), particle focusing and separation were most effective within the FR ratio range of 4–8 (Figure S6C). To minimize CTC loss and facilitate sample concentration for downstream analysis, the inner outlet resistance was increased, and the FR ratio was ultimately set to 6 (Figure 3C).

#### 3.1.3. Validation of Optimized Conditions Using Cell Lines

To validate that the flow conditions optimized with microspheres are applicable to CTC separation, verification experiments were performed using the FDMC sample dilution protocol (diluting whole blood to HCT = 4%) [46], in which Mahlavu cells (10^5^ cells·mL^−1^) were spiked into the diluted samples. The cell spiking procedure is described in Supplementary Methods S8. Since the primary spiral serves to prefocus CTCs and remove most blood cells, whereas the secondary spiral is critical for achieving high sorting purity (the main objective of this work), the flow rate of sheath 1 was kept at its optimal value. According to Equations (S16) and (S17) in Supplementary Methods S3, cell recovery and purity were subsequently evaluated under varying sheath 2 flow rates. The method for cell identification is detailed in Supplementary Methods S11. When the $Re_{c}$ of the secondary spiral was 64, both recovery (99.2 ± 0.3%) and purity (97.1 ± 1.8%) reached their maximum values (Figure 3D,E). This result is consistent with the optimal conditions identified in the aforementioned microsphere experiments, indicating that these operating parameters are also suitable for cell separation. The quantification of blood cells collected from the target outlet is described in detail in Supplementary Methods S10.

#### 3.1.4. Optimization of Sample Dilution Protocol

Compared with the FDMC, the SDMC features a reduced chip volume and a redesigned spiral geometry; therefore, the applicable hematocrit range was re-evaluated. Under the optimized flow rates and flow resistance, whole blood was diluted to HCT = 1–8% and spiked with Mahlavu cells (10^5^ cells·mL^−1^) to assess recovery and purity (Figure 3F). Recovery and purity showed no significant variation within HCT = 1–4%, although excessively low HCT prolonged processing time (Figure 3G). Considering both throughput and accuracy, HCT was therefore fixed at 4%, which provided an appropriate balance among throughput, recovery, and reliability.

Under these conditions, the SDMC processed 16 mL of sample (HCT = 4%), and a recovery volume of 7.1 ± 0.5 mL was collected from the target outlet (Figure 3H), thus achieving effective sample concentration. In contrast, a recovery volume of 22.8 ± 1.6 mL was collected from the corresponding outlet of the FDMC for the same 16 mL input. Similarly, the ASDMC processed the same volume of sample and collected a target outlet fraction nearly identical to that of the SDMC, while achieving a fourfold increase in processing throughput. The fractions collected from the target outlet consisted of the original sample as well as sheath 1 and sheath 2 fluids; therefore, the total collected volume could exceed the initial sample volume. In the aforementioned and subsequent experiments, the reported recovery and purity of Mahlavu cells sorted by the SDMC under optimal flow and sample dilution conditions were consistently obtained from the same dataset.

### 3.2. Quantitative Validation of Dean-Cycle Phase Regulation in the Purification Stage

**Figure 4:**
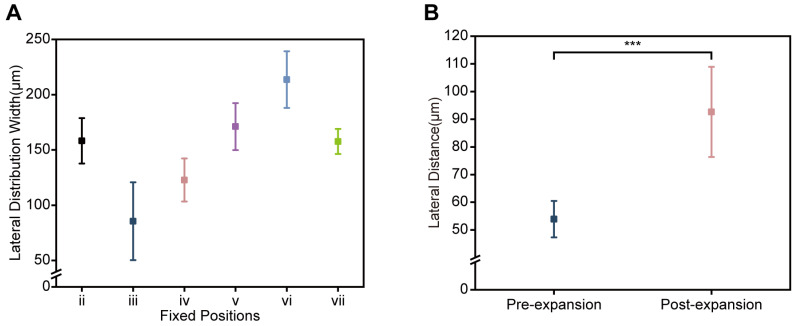
Quantitative readouts of blood-cell stream width and CTC–blood cell separation in the purification stage. (**A**) Lateral distribution width of blood cells measured at six fixed positions (ii–vii, Figure S3B) along the purification stage under optimized conditions. Data are mean ± SD from 5 independent chip sorting runs. (**B**) CTC–blood cell lateral distance measured immediately before (end of Section II) and after (onset of Section III) channel-width expansion at position vii. Data are mean ± SD from 5 independent chip sorting runs. Statistical significance: NS, not significant; *** *p* < 0.001.

At six fixed positions along the purification stage (ii–vii, as labeled in Figure S4B), we quantified the lateral distribution width of blood cells under the optimized conditions (Figure 4A). The measurements indicate that blood cells complete the first ~0.5 Dean cycle within the upstream segment spanning i–iii, and the stream width reaches its minimum at position iii, consistent with the most synchronized migration after the first half-cycle. Downstream of position iii (iii–vii), as blood cells traverse the remaining ~0.5 Dean cycle and the curvature radius increases, the lateral distribution width shows an overall increasing trend and remains broad near position vii, where blood cells are expected to approach ~1.0 Dean-cycle completion, reflecting weakened secondary flow and residual phase lag.

Because position vii corresponds to the junction between the secondary spiral (Section II) and the terminal straight segment (Section III) (as labeled in Figure S4B), we further quantified the lateral separation between CTCs and blood cells immediately before and after channel expansion (Figure 4B). Widening the channel from 400 μm to 500 μm increased the CTC–blood cell lateral distance from 53.9 ± 6.6 μm (pre-expansion, end of Section II) to 92.6 ± 16.3 μm (post-expansion, onset of Section III). This increase is consistent with an inward shift in the CTC equilibrium position induced by the aspect-ratio change [28], while the blood-cell lateral position is only weakly affected. Together, the measurements in Figure 4A,B show that section-wise geometry programming reduces blood-cell stream width in the early half-cycle, and that terminal width expansion significantly increases the CTC–blood cell lateral separation near the nominal ~1.0 Dean-cycle location. These observations are consistent with our proposed mechanism of Dean-cycle phase regulation for mitigating the migration lag effect. For all quantifications in this section, measurements were performed using ImageJ/Fiji 1.54p (National Institutes of Health, Bethesda, MD, USA), with data presented as mean ± standard deviation (SD). The lateral distribution width of blood cells was defined as the cross-channel distribution width of blood cells in high-magnification micrographs captured at each fixed position. This parameter was quantified from 5 independent chip sorting runs, defined as independent biological replicates using freshly prepared whole blood samples without spiked tumor cells. The CTC–blood cell lateral distance was defined as the linear gap between the focused CTC stream and the blood-cell stream across the channel cross-section. This parameter was quantified from 5 independent chip sorting runs, defined as independent biological replicates using freshly prepared whole blood samples spiked with Mahlavu cells (10^5^ cells·mL^−1^).

### 3.3. Systematic Verification of Sorting Performance for ASDMC

#### 3.3.1. Functional Validation Using Fluorescent Microspheres

**Figure 5:**
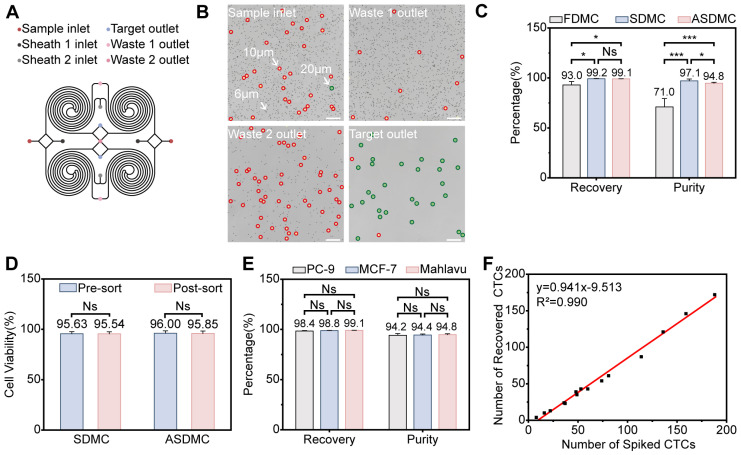
Configuration and Tumor Cell Separation Efficacy Evaluation of ASDMC. (**A**) Schematic diagram of the ASDMC structure. (**B**) Distribution of fluorescent microspheres (6 μm, 10 μm, 20 μm) after ASDMC sorting. Scale bar: 200 μm. (**C**) Comparison of recovery and purity of Mahlavu cells sorted by FDMC, SDMC, and ASDMC. Data are mean ± SD from 3 independent biological replicates per group. (**D**) Comparison of Mahlavu cell viability before and after sorting by SDMC and ASDMC. Data are mean ± SD from 3 independent biological replicates per group. (**E**) Recovery and purity of spiked PC-9, MCF-7, and Mahlavu cells sorted by the ASDMC under optimized conditions. Data are mean ± SD from 3 independent biological replicates per cell line. (**F**) Low-abundance Mahlavu cell spiking experiments in healthy blood (8–188 cells per 3 mL; *n* = 15 independent spiking and sorting runs using blood from 15 healthy donors). Statistical significance: Ns, not significant; * *p* < 0.05; *** *p* < 0.001.

Under the optimized conditions described above, ASDMC (Figure 5A) was operated at four times the flow rate of SDMC, specifically at 1200 μL·min^−1^ (sample), 2400 μL·min^−1^ (sheath 1), and 2800 μL·min^−1^ (sheath 2), while all other parameters were kept identical to those of the SDMC. The system was comprehensively evaluated under these conditions. Unless otherwise specified, “throughput” refers to the sample volumetric flow rate at the sample inlet (i.e., the sample flow rate in μL·min^−1^), excluding sheath flows.

Initial functional tests using 20 μm, 10 μm, and 6 μm fluorescent microspheres confirmed precise size-dependent separation (Figure 5B). The target outlet predominantly contained 20 μm fluorescent microspheres, accompanied by a small fraction of 10 μm fluorescent microspheres and virtually no 6 μm fluorescent microspheres, indicating that the ASDMC enables stable focusing and high-purity size separation under high-throughput parallel operation.

#### 3.3.2. Comparative Analysis of Sorting Performance

A comparative evaluation of FDMC, SDMC, and ASDMC was performed using Mahlavu cells at 10^5^ cells·mL^−1^ (Figure S7A), which revealed average recoveries of 93.0 ± 3.4%, 99.2 ± 0.3%, and 99.1 ± 0.1%, and purities of 71.0 ± 8.4%, 97.1 ± 1.8%, and 94.8 ± 0.9%, respectively (Figure 5C). Both SDMC and ASDMC achieved substantially higher recovery and purity than FDMC. The WBC depletion efficiency was determined from the fractions collected from the target outlet. All three chips exhibited target-outlet fractions with negligible RBC contamination, corresponding to RBC depletion efficiencies exceeding 99.9%. Thus, RBC depletion is not discussed further in this work. According to Equation (S18) in Supplementary Methods S3, the corresponding WBC depletion efficiencies were 67.0 ± 10.3% for FDMC, 97.9 ± 1.1% for SDMC, and 93.3 ± 0.9% for ASDMC, respectively (Figure S7B). Additionally, cell viability was assessed using Mahlavu cells at 10^5^ cells·mL^−1^ via Trypan blue staining, and showed no significant changes before and after sorting. For SDMC, viability was 95.6 ± 2.1% before and 95.5 ± 2.0% after sorting; for ASDMC, it was 96.0 ± 2.5% before and 95.9 ± 2.5% after sorting (Figure 5D). Remarkably, despite operating at a fourfold higher flow rate, ASDMC maintained comparable recovery to that of SDMC, with only a slight decrease in purity, while still preserving excellent cell viability. These results highlight the scalability and parallelization capability of ASDMC. The minor purity reduction likely stems from the dual-target outlet configuration of ASDMC, where the precise balancing of flow resistance is more challenging than in the single-target outlet of the SDMC.

#### 3.3.3. Multi-Cell-Line Validation and Low-Abundance Simulation

After confirming that ASDMC maintained SDMC-level performance at a fourfold higher throughput, we further evaluated its performance using additional tumor cell lines under the same optimized conditions. Independent spike-in experiments were performed using PC-9 and MCF-7 cells (10^5^ cells·mL^−1^), and the results were compared with the Mahlavu data obtained in Section 3.3.2. Across the three tested cell lines (Mahlavu, PC-9, and MCF-7), the mean recovery and purity were 98.8 ± 0.4% and 94.5 ± 1.1%, respectively (Figure 5E). These results suggest that the device can maintain favorable sorting performance across the tested tumor cell models. To mimic clinically relevant low-abundance CTC conditions, we performed 15 independent spike-in experiments (Table S2). In each experiment, 3 mL of healthy donor whole blood was spiked with 8–188 Mahlavu cells and then processed (Figure 5F). Recovered counts were linearly correlated with spiked counts (y = 0.941x − 9.513, R^2^ = 0.990), with a mean recovery of 73.5 ± 12.2%. This recovery was lower than that obtained in the high-concentration spike-in experiments (98.8%). The negative intercept in the linear regression model has no direct physical meaning in practical detection, as it does not imply negative cell recovery at zero spiking dose. This mathematical outcome primarily stems from intrinsic factors in low-abundance cell detection: inherent statistical fluctuations under ultra-low cell counts (e.g., 8, 16 cells per 3 mL), where target cell distribution follows a Poisson distribution leading to random deviations in the actual number of cells available for on-chip sorting; minor random errors in ImageJ-based identification and counting, which are amplified at low abundances; and inevitable absolute cell loss throughout sample processing, on-chip sorting, and downstream immunofluorescence identification. Such fixed losses have negligible impacts on high-concentration recovery but significantly affect proportional recovery at ultra-low spiking doses, ultimately manifesting as a negative intercept that reflects the performance boundary of the method under extremely low cell count conditions. We further infer that a contributing factor to the lower recovery may be a subset of tumor cells falling near or below the device’s effective size threshold—an effect more pronounced when only a small number of target cells are present, though not unique to low-abundance settings. Accordingly, the regression primarily indicates overall analytical consistency across the tested range (8–188 cells per 3 mL), rather than precise single-cell-level quantification.

#### 3.3.4. Additional Performance Assessment

Since CTCs are typically larger than blood cells—a key premise underpinning size-based enrichment—we independently measured the diameters of WBCs, RBCs, PC-9, MCF-7, and Mahlavu cells (*n* = 500 per cell type). The mean diameters were 10.2 ± 1.6 μm for WBCs, 6.9 ± 0.7 μm for RBCs, 17.1 ± 1.0 μm for PC-9, 18.0 ± 1.0 μm for MCF-7, and 19.1 ± 1.2 μm for Mahlavu (Figure S6C). To further validate size-based separation, 500 randomly selected cells collected from each outlet in the Mahlavu spiking experiments were measured after sorting. The mean diameters were 18.6 ± 2.1 μm for the target outlet, 6.9 ± 1.7 μm for waste 1 outlet, and 9.6 ± 2.2 μm for waste 2 outlet (Figure S6D).

Together, these findings demonstrate that the ASDMC achieves stable, efficient, and highly specific CTC isolation across high-throughput and ultra-low-abundance analytical settings, with exploratory feasibility confirmed in a pilot clinical cohort. These results support the further development of this scalable platform for liquid biopsy applications.

### 3.4. Application Assessment of ASDMC in Clinical Sorting of CTCs

**Figure 6:**
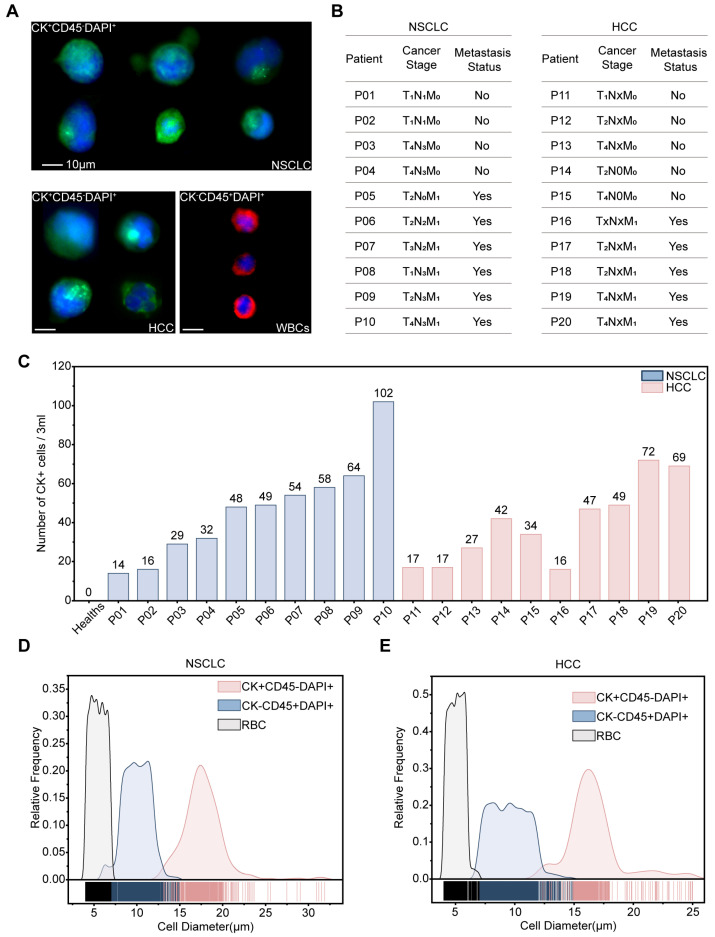
CTC Detection Analysis of ASDMC in Clinical Samples. (**A**) Representative immunofluorescence images of CTCs and WBCs from patient samples (NSCLC: *n* = 10; HCC: *n* = 10). Scale bar: 10 μm. (**B**) Clinical staging and metastasis status of NSCLC and HCC cohorts. The first column shows results from 3 healthy donor samples, in which no CTCs were detected (count = 0 for all). Each subsequent column represents an individual clinical patient sample (10 NSCLC and 10 HCC patients in total). (**C**) Enumeration of CTCs in 3 mL whole blood from all patients. (**D**,**E**) Size distributions of CTCs, WBCs (*n* = 2000 per cohort, 200 cells per patient), and RBCs (*n* = 2000 per cohort, 200 cells per patient) in NSCLC (**D**) and HCC (**E**) samples.

Having validated ASDMC using high-concentration spike-in tests across multiple tumor cell lines and by simulating ultra-low-abundance CTC conditions in healthy donor blood, we next assessed its clinical performance using peripheral blood specimens from patients with NSCLC and HCC. Detailed clinical sample processing procedures are described in Supplementary Methods S9. Immunofluorescence staining with CK, CD45, and DAPI enabled clear identification of CK^+^CD45^−^DAPI^+^ CTCs and CK^−^CD45^+^DAPI^+^ WBCs (Figure 6A). The detailed immunofluorescence staining protocols are described in Supplementary Methods S10. Clinical samples were collected from ten NSCLC patients (2 early stage, 8 late stage, including 6 with metastases) and ten HCC patients (3 early stage, 7 late stage, including 5 with metastases) (Figure 6B). Enumeration of CK^+^CD45^−^DAPI^+^ cells in 3 mL whole blood showed an exploratory trend toward higher CTC counts in patients with more advanced disease: early-stage patients showed approximately 5–10 CTCs·mL^−1^, late-stage patients 10–15 CTCs·mL^−1^, and metastatic cases generally exceeding 15 CTCs·mL^−1^ (Figure 6C). Because this pilot cohort is underpowered for formal statistical analysis of stage-related differences, these observations should be regarded as preliminary. To provide a negative-control reference, three healthy donor whole-blood samples were also processed using the same sorting and downstream identification workflow, and no CTCs were detected (Figure 6C). For cell diameter analyses, RBCs were collected from both waste 1 and 2 outlets of the ASDMC, while CK^−^CD45^+^DAPI^+^ WBCs were obtained from the waste 2 and the target outlets, ensuring accurate representation of true size distributions during sorting. Within each disease cohort (NSCLC and HCC), we measured 2000 WBCs and 2000 RBCs in total (200 cells per type per patient). In the NSCLC cohort, 466 CTCs (mean diameter: 17.8 ± 2.5 μm), 2000 WBCs (10.0 ± 1.5 μm), and 2000 RBCs (5.5 ± 0.9 μm) were analyzed (Figure 6D). In the HCC cohort, 390 CTCs (16.6 ± 2.3 μm), 2000 WBCs (9.5 ± 1.6 μm), and 2000 RBCs (5.1 ± 0.6 μm) were analyzed (Figure 6E). Overall, these findings support the feasibility of using ASDMC for CTC isolation in clinical blood samples, although larger studies will be needed to further assess its clinical utility.

## 4. Discussion

This study systematically establishes the design rationale, hydrodynamic optimization, and preliminary performance evaluation of ASDMC for label-free enrichment of CTCs from diluted whole blood. By parallel integration of four optimized SDMC units, ASDMC maintains uniform flow distribution and balanced hydraulic resistance across units, enabling a fourfold throughput increase without a significant loss in high-concentration spike-in recovery or purity. Under optimized conditions, ASDMC achieves a mean tumor cell recovery of 98.8% in high-concentration spike-in tests and a mean WBC depletion efficiency of 93.3%, with negligible RBC contamination in the target outlet fraction. To assess its preliminary clinical potential, a pilot clinical study was conducted using 20 patient samples (including NSCLC and HCC). The enriched fractions enabled immunofluorescence identification of CK^+^CD45^−^DAPI^+^ CTCs, supporting the preliminary feasibility of the platform for downstream CTC analysis.

A key feature of this work is a design strategy informed by the hypothesized blood-cell migration “lag effect” in spiral inertial microfluidics. We postulate that under high blood-cell loading, long-range hydrodynamic interactions broaden the focused blood-cell stream [48,49], and that asynchronous completion of the ~0.5 and ~1.0 Dean-cycle migrations by a subpopulation of blood cells (the lag effect) compresses the lateral CTC–blood cell separation and limits purification efficiency. Based on this hypothesis, we implemented a Dean-cycle phase regulation strategy within the double-spiral purification stage by programming the channel geometry into three functional sections. In Section I and the initial portion of Section II, the mean curvature radius is minimized to increase the Dean number in the first half-cycle, strengthening the Dean-driven secondary flow and promoting the synchronized migration of blood cells toward the inner wall, thereby reducing the fraction of cells that fail to complete ~0.5 DC on time. As the curvature radius gradually increases in the later portion of Section II, the secondary flow diminishes and some lag in the second half-cycle becomes difficult to avoid. We therefore introduce a straight Section III with an expanded channel width (400 μm to 500 μm), which increases the aspect ratio and shifts the equilibrium position of larger CTCs further toward the inner wall, while smaller blood cells—experiencing minimal inertial lift in the straight channel—retain nearly unchanged lateral positions. This design enlarges the CTC–blood cell gap near one full Dean cycle and improves overall separation efficiency. The agreement between theoretical analysis and experimental observations supports this mechanistic interpretation and highlights the gentle yet efficient nature of inertial microfluidic enrichment. We acknowledge that the current observations do not provide direct, single-particle-level validation of migration phase synchronization, as the stream width changes could also be partially attributed to enhanced Dean-driven secondary flow in the reduced-curvature-radius segments. Direct tracking of individual particle trajectories via high-speed imaging would be required in future work to definitively verify the proposed phase regulation mechanism and quantify the relative contributions of each geometric design element to the final separation performance.

We also incorporated a balancing channel to improve inter-stage hydrodynamic decoupling, which we consider important for maintaining stable operation in the cascaded spiral design. Previous studies by Agnihotri et al. suggested that adaptive resistance tuning through branch or bypass channel geometry optimization can help reduce upstream pressure feedback and improve flow partitioning stability in cascaded microfluidic systems [30]. Our results are generally consistent with this idea: under the optimized balancing-channel configuration, flow rate fluctuations between stages were reduced, which likely helped maintain more consistent hydrodynamic conditions for cell focusing in the two spiral segments. Compared with conventional single-spiral [53] or slanted-spiral [54] microchannels, as well as our previously reported FDMC [46], the SDMC/ASDMC platform provides a practical balance among throughput, recovery, WBC depletion efficiency and cell viability under the tested operating conditions.

Beyond single-channel optimization, the parallel array design of ASDMC addresses a key challenge in inertial microfluidics: scaling throughput for clinical translation without compromising separation performance. The development of parallelized, high-throughput microfluidic platforms has been a major focus of rare-cell sorting research in recent years. Fachin et al. developed a monolithic microfluidic chip for high-throughput blood cell depletion and rare CTC sorting, while Chen et al. reported a triplet parallel spiral chip for continuous tumor cell separation [55,56]. Jeon et al. further advanced this field with a fully automated, field-deployable blood separation platform based on multi-dimensional double spiral inertial microfluidics [57,58,59]. Our work builds on this engineering progression from single-unit proof-of-concept to scalable arrayed systems. Notably, ASDMC maintains uniform flow distribution and balanced hydraulic resistance across all four parallel units using only three independently controlled syringe pumps, eliminating the need for additional pumping hardware for each channel. This design simplifies operation and supports practical clinical deployment, while retaining the high separation performance of the single SDMC unit.

Despite these advances, several challenges remain. First, the current system requires low hematocrit to avoid clogging and maintain focusing stability; direct processing of undiluted whole blood remains a key objective for future development. Second, compared with the single-target-outlet SDMC, the ASDMC exhibited a slight reduction in purity, most likely due to the dual-target-outlet configuration in the secondary spiral, in which maintaining identical flow resistance between two target outlets is inherently more difficult. Third, the present readout relies on immunofluorescence imaging, which introduces additional handling steps and may interfere with certain downstream single-cell analyses. Fourth, the limited sample size of the current pilot clinical cohort only supports preliminary feasibility assessment of the platform, and further validation in a larger clinical cohort is required to verify the clinical utility of this device. Fifth, the heuristic $R_{f}$ criterion adopted for stable focusing in this work is an engineering guideline rather than an absolute physical threshold. We acknowledge that stable focusing may be achieved under conditions with $R_{f}$ close to 1, while instability may still occur at $R_{f}\geq 1$ under high cell loading or non-ideal flow conditions. This criterion is solely used for initial geometric parameter screening in this work, and a refined theoretical model incorporating actual cell loading and flow conditions is needed for more precise channel optimization in future studies. These limitations could be mitigated through the integration of on-chip, real-time detection modules—such as impedance-based cytometry or label-free optical sensing—to enable highly automated classification and enumeration directly after enrichment. In addition, combining the ASDMC with single-cell sequencing and live-cell culture systems would further enhance its clinical translational value and support its application in precision oncology.

In summary, the ASDMC provides a scalable inertial microfluidic platform for high-throughput, label-free CTC enrichment from clinically relevant blood volumes under the tested experimental conditions. By introducing Dean-cycle phase regulation to suppress blood-cell migration lag, this work offers a mechanism-guided route to improving purification performance while preserving cell integrity, laying a foundation for further development of downstream diagnostic and translational applications.

## 5. Conclusions

We developed a four-channel inertial microfluidic array (ASDMC) for label-free enrichment of CTCs from diluted whole blood. By proposing a design strategy targeting the hypothesized blood-cell migration “lag effect” via Dean-cycle phase regulation, we developed a platform that achieves high-throughput CTC purification under the tested conditions. The observed separation performance is consistent with the proposed mechanism of promoting early-cycle blood-cell migration synchrony and widening the CTC–blood cell gap near one Dean cycle. At HCT = 4%, ASDMC achieves a sample throughput of 1200 μL·min^−1^ while maintaining high recovery (98.8 ± 0.4%) in high-concentration spike-in tests and WBC depletion efficiency (93.3 ± 0.9%), yielding an enriched target fraction of ~7 mL from a 16 mL input. Pilot testing in 20 patient samples (NSCLC/HCC) supports the preliminary feasibility of the platform for CTC enumeration in clinical samples and provides a basis for further evaluation in larger cohorts. Future work will focus on higher-hematocrit operation, integrated on-chip detection, and further clinical validation of the platform.

## Data Availability

The original contributions presented in this study are included in the article/Supplementary Materials. Further inquiries can be directed to the corresponding author.

## Supplementary Materials

The following supporting information can be downloaded at: https://www.mdpi.com/article/10.3390/mi17040446/s1, including Supplementary Methods S1–S11, Figures S1–S7, and Tables S1 and S2. References [28,50,51,52,60,61,62,63,64,65] are cited in supplementary file.

## Abbreviations

The following abbreviations are used in this manuscript:

CTC(s)	Circulating Tumor Cell(s)
SDMC(s)	Second-generation Double-Spiral Microfluidic Chip(s)
ASDMC	Arrayed Second-generation Double-Spiral Microfluidic Chip
HCT	Hematocrit
NSCLC	Non-Small Cell Lung Cancer
HCC	Hepatocellular carcinoma
CK	Cytokeratin
CD45	Cluster of differentiation 45
DAPI	4′,6-diamidino-2-phenylindole
FDMC	First-generation Double-Spiral Microfluidic Chip
DC	Dean cycle
RBC(s)	Red Blood Cell(s)
WBC(s)	White Blood Cell(s)
FR	Flow resistance
