# Supplementary material for: Suppressing Blood-Cell Migration Lag via Dean-Cycle Phase Regulation Enables High-Purity CTC Enrichment in an Inertial Microfluidic Array
Source: Micromachines (Basel). 2026 Apr 3;17(4):446. doi: 10.3390/mi17040446 (PMC13118628; doi:10.3390/mi17040446)
Supplement: Supplementary file 1 [file micromachines-17-00446-s001.zip › micromachines-4216056-supplementary.pdf]

Supplementary Material

Suppressing Blood-Cell Migration Lag via Dean-Cycle Phase Regulation Enables High-Purity CTC Enrichment in an Inertial Microfluidic Array

Tai-Hang Wu [1,2,†], Hao-Zheng Li [1], Xian-Ge Sun [1], Xiao-Dong Ren [1], Hong Wang [1] and Qing Huang [1,2,*]

[1] Department of Laboratory Medicine, Daping Hospital, Army Medical University, Chongqing, 400042, China
[2] Department of Biomedical Materials Science, Army Medical University, Chongqing 400038, China
* Correspondence: qinghuang@tmmu.edu.cn
† Tai-Hang Wu is the sole first author of this work.

Content

Supplementary Methods S1. Archimedean Spiral Geometry 2
Supplementary Methods S2. Inertial Focusing Theory 2
Supplementary Methods S3. Formulas for efficiency evaluation 4
Supplementary Methods S4. COMSOL Multiphysics Numerical Simulation Setup 4
Supplementary Methods S5. Fabrication 5
Supplementary Methods S6. Preparation of fluorescent microspheres 5
Supplementary Methods S7. Flow distribution and flow resistance characterization 6
Supplementary Methods S8. Cells and Cell Culture 6
Supplementary Methods S9. Preparation of clinical samples 6
Supplementary Methods S10. Immunofluorescence Staining 6
Supplementary Methods S11. ImageJ-Based Cell Enumeration 7
Table S1. Core fluid physical properties for COMSOL simulations 8
Table S2. Sample-level data of low-abundance spike-in experiments 9
Supplementary Figures 10
Supplementary Bibliography 15

Supplementary Methods S1. Archimedean Spiral Geometry

Each spiral microchannel in this study was designed in the form of an Archimedean spiral, described mathematically as [1]:

$$r = a + b\theta \quad \text{(S1)}$$

$$b = \frac{d}{2\pi} \quad \text{(S2)}$$

where r denotes the radial radius, a is the initial radius of the spiral, and b is a parameter that controls the rate at which the spiral increases. Specifically, b determines how much the r changes for each unit increase in the angular position (θ). It is directly related to the pitch (d) of the spiral. The d is one of the geometric characteristics of the spiral, representing the radial distance the spiral increases for one full revolution (i.e., 2π angle). The primary spiral was positioned on the outer side relative to the center of the chip, while the secondary spiral was located on the inner side. In this study, the primary and secondary spirals were constructed with $a_1 = 3.2\ \text{mm}$ and $a_2 = 2.3\ \text{mm}$, respectively, and each channel contained 1.75 turns; however, their initial angular positions (θ_i) were independently set, allowing for precise adjustment of the channel geometry. Due to the compact size of the chip and to ensure the stability of subsequent bonding, a pitch of 3.7 mm was selected.

Supplementary Methods S2. Inertial Focusing Theory

When fluid flows through a curved microchannel, centrifugal forces generate two counter-rotating Dean vortices near the channel walls[2]. The strength of these Dean vortices is characterized by the Dean number (De), which quantifies the combined influence of channel curvature and the primary-flow inertia [3,4]:

$$De = Re_c \left(\frac{D_h}{2R} \right)^{1/2} \quad \text{(S3)}$$

R is the channel curvature radius, defined as:

$$R = \frac{\left(r^2 + b^2\right)^{3/2}}{r^2 + 2b^2} \quad \text{(S4)}$$

The mean curvature radius (R_{avg}) over the spiral segment $[\theta_i, \theta_{end}]$ is defined in the arc-length sense as:

$$R_{avg} = \frac{\int R ds}{\int ds} \quad \text{(S5)}$$

where $ds = \sqrt{r^2 + \left(\frac{dr}{d\theta}\right)^2} d\theta$. Since each spiral consists of 1.75 turns, determining the initial angular position (θ_i) is sufficient to compute the ending angular position (θ_{end}). In the calculation of the De, R_{avg} was used to represent the overall curvature of the spiral channel, as it provides a more accurate characterization of the channel's geometric properties over the entire angular range.

And Re_c is the channel Reynolds number [4], defined as:

$$Re_c = \frac{\rho U_m D_h}{\mu} \quad \text{(S6)}$$

where the hydraulic diameter (D_h) is given by $D_h = (2wh) / (w+h)$, w and h are the

channel width and height, respectively, ρ denotes the fluid density (kg·m^{-3}), and μ is the dynamic viscosity (Pa·s). The maximum flow velocity (U_m) is related to the average flow velocity (U_f) [2] as:

$$U_f = \frac{Q}{A} \tag{S7}$$

$$U_m = 2U_f \tag{S8}$$

where Q denotes the volumetric flow rate (m^3 s^{-1}), and A is the cross-sectional area (m^2) [2].

Within such curved channels, suspended particles are mainly subjected to two hydrodynamic forces: the inertial lift force (F_L) and the Dean drag force (F_D). The inertial lift force can be expressed as [5]:

$$F_L = \frac{\rho U_m^2 \alpha_p{}^4}{D_h^2} f_L(Re_c, x_C) \tag{S9}$$

where α_p is the particle diameter, and the lift coefficient $f_L(Re_c, x_C)$ is a function of the position of the microparticle within the cross-section of the microchannel (x_C) and the channel Reynolds number (Re_c) [6,7]. For channels with a rectangular cross-section and Re_c below 100, the lift coefficient takes a value of 0.5 in this work as a widely used engineering approximation for preliminary design and theoretical estimation [2,7]. We acknowledge that this simplification does not capture the spatial variation of $f_L(Re_c, x_C)$ across the channel width, and thus only provides a first-order estimation of inertial lift force, rather than a precise description of position-dependent particle dynamics. The Dean drag force arises from particle entrainment in the counter-rotating Dean vortices, and its magnitude is governed by Stokes' law for low-Reynolds-number particle motion relative to the secondary flow [2,3]:

$$F_D = 3\pi\mu U_{Dean}\alpha_p \tag{S10}$$

$$U_{Dean} = 1.8\times10^{-4} De^{1.63} \tag{S11}$$

Here, U_{Dean} denotes the characteristic velocity of the Dean secondary flow. U_{Dean} scales with the Dean number (De) via the aforementioned empirical correlation, which describes the cross-section-averaged velocity of the secondary flow within the channel. It should be explicitly noted that this correlation only provides a bulk-averaged estimation, and cannot capture the spatial heterogeneity of Dean vortices across the channel height and width. In this work, the coupling between De, the secondary flow field, and particle migration is described semi-quantitatively to guide the geometric design of the channel, rather than to perform fully resolved numerical simulations of single-particle trajectories.

The particle motion in the channel is determined by the competition between F_L and F_D. Large particles experiencing strong F_L focus near stable equilibrium positions, while small particles dominated by F_D circulate within the Dean vortices [2,7]. To quantify their relative contributions, the ratio R_f [5] is defined as:

$$R_f = \frac{F_L}{F_D} \sim \frac{1}{\delta}\left(\frac{\alpha_p}{D_h}\right)^3 Re_c^n \qquad (n<0) \tag{S12}$$

The curvature ratio is $\delta = D_h/(2R)$ [5]. For cross-sectional dimension selection in spiral microchannel design, the inertial focusing criterion $R_f = 1$ is adopted [8]. When $R_f \geq 1$, inertial lift dominates and stable focusing occurs; when $R_f < 1$, Dean drag dominates and particles migrate along the Dean vortices [8]. The stable focusing condition also requires the particle confinement ratio $\alpha_p / D_h > 0.07$ [2,5,7].

The formula for the lateral migration velocity of the particle (U_L) is expressed as [9,10], where $C_L = f_L(Re_c, x_C)$:

$$U_L = \frac{\rho U_{\mathrm{m}}^2 \alpha_p{}^3 C_L}{3\pi\mu D_h^2} \tag{S13}$$

The migration lengths required for a 20 μm particle to focus (L_I) and a 10 μm particle to complete one Dean cycle (L_D) are as follows [2,3,7,10], where $L_M = w$:

$$L_I = \frac{U_f}{U_L} \times L_M \tag{S14}$$

$$L_D = \frac{U_f}{\bar{U}_{\mathrm{Dean}}} \times L_M \tag{S15}$$

Here, $\bar{U}_{\mathrm{Dean}}$ represents the average value of the Dean vortices velocity over a given region or period, indicating the typical speed induced by the Dean effect in the flow [10].

This framework guided the geometric and hydrodynamic design of the SDMC and ASDMC devices used in this study.

Supplementary Methods S3. Formulas for efficiency evaluation

System efficiency was evaluated by measuring recovery, purity, throughput (volume per unit time), WBC depletion efficiency, and viability (via Trypan Blue exclusion assay). Recovery and purity were calculated using the final cell counts obtained after sorting, collection, centrifugation, staining, and microscopic imaging-based counting. Specifically, N_{input} denotes the number of target cells spiked into the sample inlet, N_{target} denotes the number of target cells counted in the collected target fraction, and $N_{\mathrm{recovered}}$ denotes the total number of cells counted in the collected target fraction (target cells plus background blood cells). In addition, $N_{\mathrm{WBC,target}}$ denotes the total number of WBCs introduced into the sample inlet, and $N_{\mathrm{WBC,inlet}}$ denotes the number of WBCs counted in the collected target fraction from the target outlet. Recovery, purity, and Depletion efficiency were calculated as:

$$\mathrm{Recovery} = \frac{N_{\mathrm{target}}}{N_{\mathrm{input}}} \times 100\% \tag{S16}$$

$$\mathrm{Purity} = \frac{N_{\mathrm{target}}}{N_{\mathrm{recovered}}} \times 100\% \tag{S17}$$

$$\mathrm{WBC\ Depletion\ efficiency} = \left(1 - \frac{N_{\mathrm{WBC,target}}}{N_{\mathrm{WBC,inlet}}}\right) \times 100\% \tag{S18}$$

Supplementary Methods S4. COMSOL Multiphysics Numerical Simulation Setup

This section details the steady-state laminar flow simulations performed in COMSOL Multiphysics 6.3 (COMSOL Inc., Sweden). These simulations were conducted to optimize the inter-stage hydrodynamic decoupling structure and balancing channel width. The results provided core design guidance for subsequent device fabrication. However, particle tracking or particle dynamics modeling was not included in these fluid-only simulations. The cross-sectional dimensions of the primary and secondary spiral channels were first optimized using MATLAB simulations. Based on this, we constructed the geometry of the primary and secondary spiral channels in SolidWorks version 2022 (Dassault Systèmes SolidWorks Corporation, USA). The transition region between the two spirals was set as an undefined area with variable curvature, initially filled with an arbitrary configuration. The complete 3D fluidic model was imported into

COMSOL multiphysics via the LiveLink for SOLIDWORKS interface.

All simulations were based on the steady-state incompressible laminar flow module (denoted as spf in the software). The flow was governed by the steady Navier-Stokes equations and the continuity equation under isothermal conditions at 25 °C, with no heat or mass transfer effects considered in the computational domain. The boundary conditions for the simulations were as follows: a no-slip boundary condition was applied to all solid walls of the microchannels. Fully developed laminar flow with a fixed volumetric flow rate was used for all inlets. The sample inlet flow rate was set at 300 $\mu L \cdot min^{-1}$, and the sheath 1 inlet flow rate was set at 500 $\mu L \cdot min^{-1}$. An auxiliary parametric sweep was performed for the sheath 2 inlet flow rate, ranging from 300 to 700 $\mu L \cdot min^{-1}$ in increments of 100 $\mu L \cdot min^{-1}$ to evaluate the system's flow stability. A static pressure outlet boundary condition with a reference pressure of 0 Pa was applied to all outlets. The backflow suppression function was enabled at all outlets to eliminate non-physical reverse flow and ensure the stability of the numerical solution. To identify the optimal balancing channel width for inter-stage hydrodynamic decoupling, a parametric sweep for the balancing channel width was performed in the range of 270–310 μm. The standard deviation of the flow rate ratio between the primary CTC outlet and the waste 1 outlet was quantified across all tested sheath 2 flow conditions. A lower standard deviation indicated better hydrodynamic decoupling between the two spiral stages.

All simulations were calculated using a steady-state solver paired with the built-in direct linear solver of the software. The relative convergence tolerance was set to 0.001. Only the laminar flow physics interface was included in the solver contribution terms. A physics-controlled mesh with a "Finer" element size was applied to the entire computational domain. Automatic mesh refinement was used near the channel walls and in regions with large velocity gradients, such as curved spiral segments, cross-section transition regions, and the balancing channel. The fluid used in the simulations was defined as phosphate-buffered saline (PBS), with all physical properties calculated at a constant temperature of 25 °C. Detailed core parameters are listed in Table S1.

Supplementary Methods S5. Fabrication

The SDMC and ASDMC were designed in-house using SolidWorks 2022 (Dassault Systèmes, France) and exported to AutoCAD 2020 (Autodesk, USA) format for mask layout. Fabrication was outsourced to Hanguang Co. (China) using standard soft-lithography procedures. Briefly, degassed polydimethylsiloxane (PDMS; Sylgard 184, Dow Corning Inc., USA) was prepared by mixing base and curing agent at a 10:1 (w/w) ratio, cast onto a master mold, and cured at 80 °C for 1 h. After curing, the PDMS replica was released from the mold, and all inlet/outlet holes were pre-punched by the manufacturer. The PDMS layer was then irreversibly bonded to a glass substrate by oxygen-plasma activation to form enclosed microchannels. All devices used in this study were supplied as fully assembled chips by Hanguang Co. (China), and no additional on-site punching or bonding steps were required.

Supplementary Methods S6. Preparation of fluorescent microspheres

Three sizes of fluorescent polystyrene microspheres were employed to model major blood cell types under flow. Specifically, 6 μm microspheres (2.1×10^8 particles$\cdot mL^{-1}$; Aladdin, China) imitated RBCs; 10 μm (3.6×10^7 particles$\cdot mL^{-1}$; Spherotech, USA) and 20 μm (4.0×10^6 particles$\cdot mL^{-1}$; Spherotech, USA) microspheres represented WBCs and CTCs, respectively. All microsphere suspensions were diluted with phosphate-buffered saline (PBS; HyClone, USA) containing 0.1% v/v Tween-20 (SIGMA, USA) to prevent particle sedimentation and 0.5% v/v bovine serum albumin (BSA; SIGMA, USA) to reduce nonspecific adhesion to microchannel walls.

Supplementary Methods S7. Flow distribution and flow resistance characterization

We combined experiments and simulations to quantify flow distribution and tune flow resistance in the cascaded-spiral device. Laminar-flow simulations were performed in COMSOL. In parallel, particle motion was recorded using a high-speed camera (FASTCAM NOVA S9, Photron, Japan) mounted on an inverted microscope (Olympus IX73, Olympus Corporation, Japan). Particle velocities were obtained by tracking trajectories in the recorded videos. The volumetric flow rates at the primary CTC outlet, target outlet, and waste 2 outlet were estimated as $Q = v \times A$, where v is the measured particle velocity and A is the channel cross-sectional area. Under steady laminar flow with stable inlet pressure, and when the measured channel segment dominates the flow resistance, the estimated Q reliably represents the local fluid flow rate. These measurements were used to calculate channel flow resistances and were cross-checked against COMSOL predictions to support device optimization.

Supplementary Methods S8. Cells and Cell Culture

Three human cancer cell lines (PC-9, MCF-7, and Mahlavu) were used in this study. Based on Cellosaurus records, PC-9 is a human lung adenocarcinoma cell line established from a metastatic lymph node (male donor, 45 years; RRID: CVCL_B260), MCF-7 is a human invasive breast carcinoma cell line established from metastatic pleural effusion (female donor, 69 years; RRID: CVCL_0031), and Mahlavu is a human hepatocellular carcinoma cell line established from liver (female donor; RRID: CVCL_0405).

Cells were maintained under standard culture conditions (37 °C, humidified atmosphere with 5% CO_2). PC-9 cells were cultured in RPMI-1640 (BasalMedia, China), whereas MCF-7 and Mahlavu cells were cultured in DMEM (HyClone, USA). For spike-in experiments, cells were harvested by centrifugation at 300 g for 5 min, collected, and resuspended in culture medium lacking fetal bovine serum (FBS; Gibco, USA). Mahlavu cells stably expressed GFP, while MCF-7 and PC-9 cells were labeled with CellTracker Green (Thermo Fisher Scientific, USA) prior to spike-in experiments.

All cultures used in this study were experimentally confirmed to be free of contamination, including being mycoplasma-negative, during routine cell culture.

Supplementary Methods S9. Preparation of clinical samples

Peripheral blood samples from NSCLC patients, HCC patients, and healthy donors were collected in EDTA tubes under institutional ethical approval (Ethics Committee of the Army Medical Center, No. 2025-092) with written informed consent. Prior to microfluidic processing, samples were diluted to a target hematocrit of ~4% and processed without RBC lysis or antibody labeling.

Supplementary Methods S10. Immunofluorescence Staining

The fractions collected from the target outlet were supplemented with 5% poloxamer (Mengbio, China) to achieve a final poloxamer concentration of 1% (based on unpublished data), and then centrifuged at 300 g for 10 min. After removing the supernatant and leaving 200 μL, the cell suspension was mixed thoroughly and dropped onto adhesion microscope slides (Beyotime, China) for subsequent immunofluorescence staining. Immunofluorescence staining was performed using a commercial kit (Dowobio, China) according to the manufacturer's protocol. Briefly, cells were fixed for 30 min and blocked for 30 min at room temperature. The cells were then incubated with primary antibodies — Cytokeratin (Pan) Mouse mAb (Immunoway, USA) and CD45 (PT0471R) PT™ Rabbit mAb (Immunoway, USA) — for 1–2 h, followed by incubation with a secondary antibody for 1 h. Nuclear counterstaining was performed with DAPI for 5 min.

For spike-in tumor cell experiments, only DAPI nuclear staining and CD45 immunofluorescence staining were required; for clinical patient samples, a full three-marker panel including cytokeratin (CK), CD45, and DAPI was used for staining. Finally, the slides were mounted and systematically scanned using a Leica DMi8 (Leica Microsystems, Germany) fluorescence microscope to acquire whole-slide images. Cell numbers of WBCs, RBCs, and CTCs were then quantified separately from the acquired images using ImageJ software (National Institutes of Health, USA). Except for the primary antibodies, all other reagents were obtained from Dowobio (Shanghai, China). CTCs were defined as $CK^+CD45^-DAPI^+$, and WBCs as $CK^-CD45^+DAPI^+$.

Supplementary Methods S11. ImageJ-Based Cell Enumeration and Quantitative Data Analysis

For the enumeration of spiked tumor cells (PC-9, MCF-7 and Mahlavu) in spike-in experiments, whole-slide fluorescence scanning was performed on the stained slides prepared as described in Supplementary Methods S10 using a fluorescence microscope, and the acquired whole-slide images were imported into ImageJ v1.54f for subsequent analysis. First, the multi-channel fluorescence images were split into individual single-channel images via the Image > Color > Split Channels function, then each single-channel image was processed sequentially with the Process > Filters > Gaussian Blur function with a Sigma value of 2.00, followed by the Image > Adjust > Threshold function with the Auto algorithm selected and the Apply operation executed, and then the Process > Binary > Make Binary and Process > Binary > Watershed operations were performed on each binary image in turn. Next, the Process > Image Calculator function was used to perform logical AND operations on the processed binary images, with the Operation set to AND, the first calculation using the red channel (representing WBCs) and blue channel as Image 1 and Image 2 respectively, and the second calculation using the green channel (representing CTCs) and blue channel as Image 1 and Image 2 respectively. The two resulting images from the AND operations were processed again via the Image > Adjust > Threshold function, and then the Analyze > Analyze Particles function was performed on each image with the Size parameter set to 10-Infinity and the Circularity parameter set to 0.3–1.0, and the total number of cells was the sum of the cell counts obtained from the two images via the Analyze Particles function. For clinical samples, after the identification of CTCs was completed via manual review, images of the confirmed CTCs were imported into ImageJ for cell size measurement, and the enumeration of CTCs in clinical samples was performed via manual microscopic counting.

Table S1. Core fluid physical properties for COMSOL simulations

Physical Property	Symbol	Value at 25 °C	Unit
Dynamic viscosity	μ	8.9×10−4	Pa·s
Density	ρ	997	kg/m³
Isobaric specific heat	C_p	4180	J/(kg·K)
Thermal conductivity	k	0.61	W/(m·K)
Electrical conductivity	σ	5.5×10−6	S/m

Table S2. Sample-level data of low-abundance spike-in experiments

Experiment No.	Number of spiked CTCs	Number of recovered CTCs	Recovery (%)
1	8	4	50.0
2	16	10	62.5
3	22	13	59.1
4	36	24	66.7
5	37	23	62.2
6	48	39	81.3
7	49	35	71.4
8	53	43	81.1
9	60	43	71.7
10	74	54	73.0
11	81	61	75.3
12	114	87	76.3
13	136	121	89
14	159	146	91.8
15	188	172	91.5

Supplementary Figures

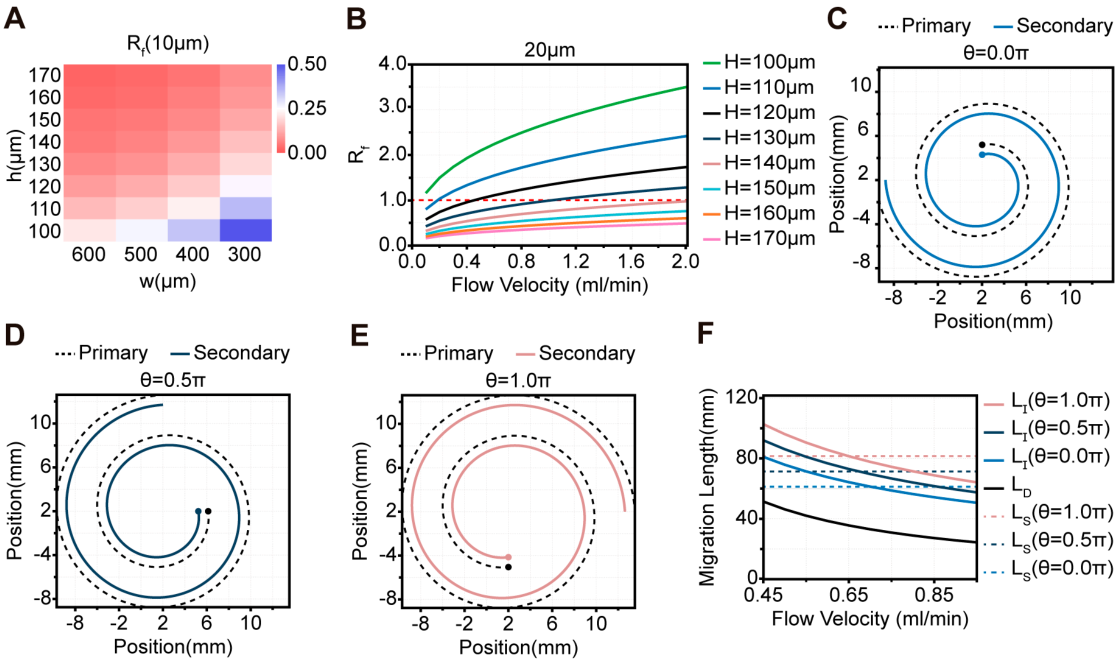

Figure S1.
(A) Simulation of the R_f for 10 μm particles at a flow rate of 800 μL·min^{-1} under different channel cross-sectional dimensions. (B) Variation of R_f with flow rate for multiple channel widths (w) at fixed height (h) for 20 μm particles. (C-E) Plots of the primary and secondary spiral centerlines at different initial angular positions (θ_i). (F) Variation of the migration length required for 20 μm particles to achieve focusing (L_I) and the migration length required for 10 μm particles to complete one Dean cycle (L_D) in the secondary spiral at different θ_i, as a function of flow rate. The arc length for 1.75 turns (L_S) is also shown for each θ_i as a reference.

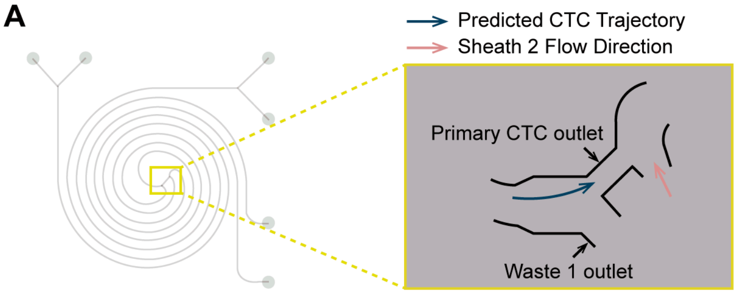

Figure S2.
(A) Schematic showing the direct connection between the primary CTC outlet and the sheath 2 channel.

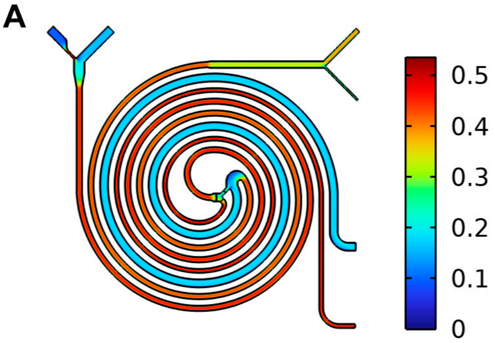

Figure S3.
(A) Representative COMSOL fluid simulation result. Balancing channel width: 290 μm, sample flow rate: 300 μL·min^{-1}, sheath 1 and sheath 2 flow rates: both 500 μL·min^{-1}.

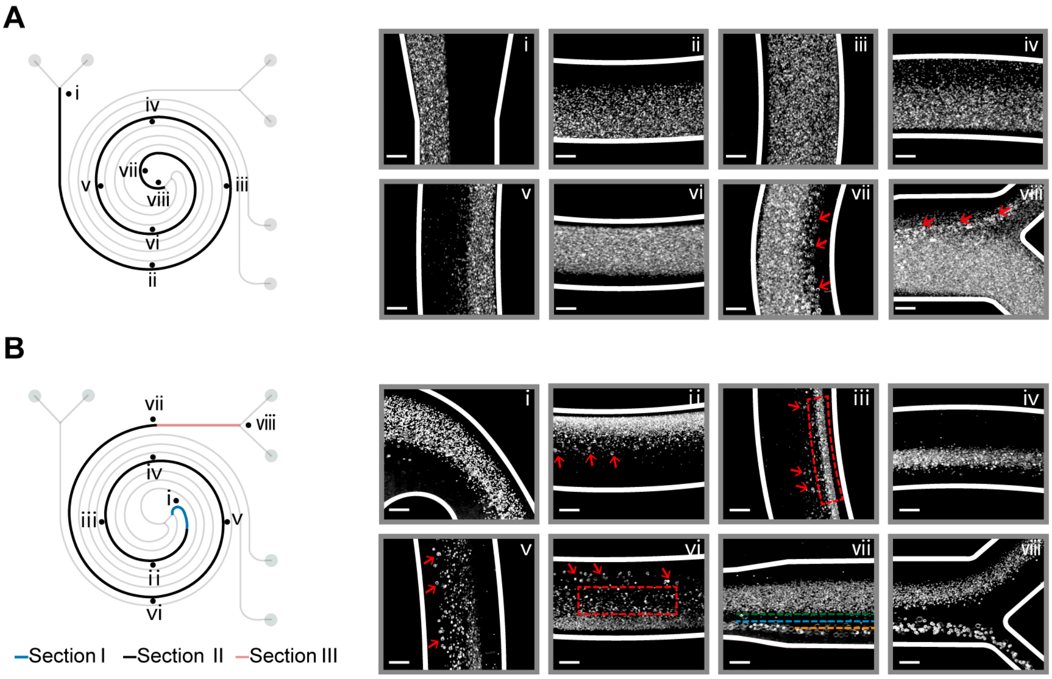

Figure S4.
(A) Schematic of the coarse separation stage in the SDMC, primarily comprising the primary spiral, which removes most RBCs and partially removes WBCs before final purification. Scale bar: 100 μm. The red arrows indicate the trajectories of CTCs. (B) Schematic of the SDMC purification stage, illustrating three functional sections for regulated secondary flow and enhanced separation of CTCs and blood cells. Inset (iii): Experimental image showing suppression of the lag effect when blood cells complete 0.5 Dean cycle (DC) (highlighted by the red dashed box). Inset (vi): Lag effect of blood cells during 0.5–1.0 DC (red dashed box). Inset (vii): Blue and yellow dashed lines indicate CTC trajectories at the end of Section II and in Section III, respectively, and the green dashed line indicates the blood cell trajectory in the same regions. Red arrows indicate CTC trajectories. Scale bar: 100 μm.

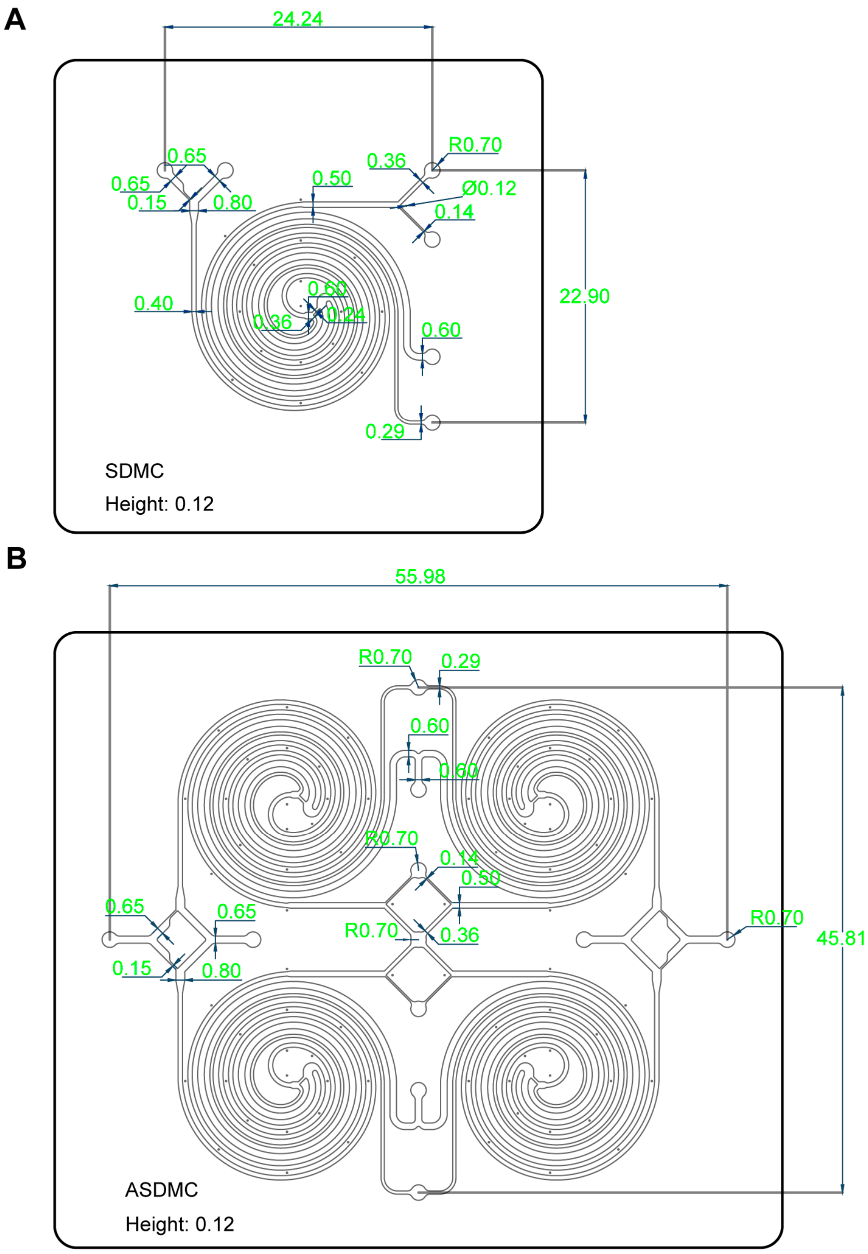

Figure S5.
(A–B) Detailed dimensional parameters of the SDMC (A) and ASDMC (B) are indicated. All parameters are in millimeters (mm).

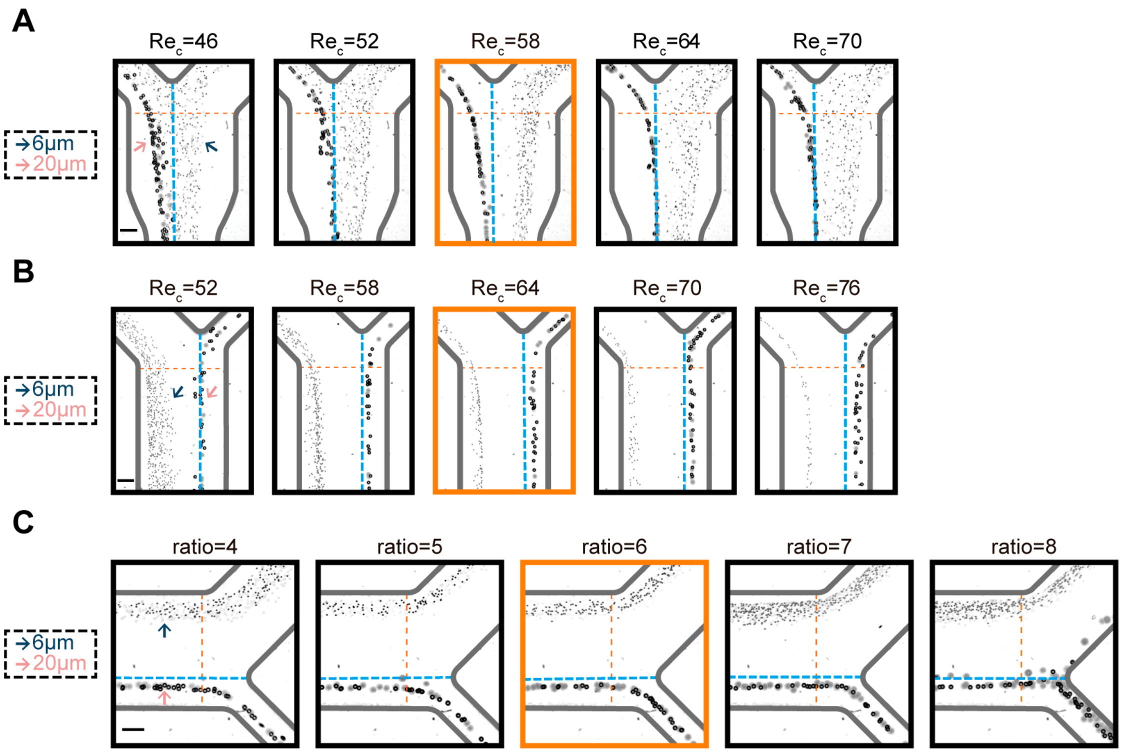

Figure S6.
(A–B) Images showing particle distribution in the primary (A) and secondary (B) spiral channels at different flow rates. Scale bar: 100 μm. (C) Images illustrating the effect of flow resistance on particle focusing behavior in the secondary spiral channel. Scale bar: 100 μm.

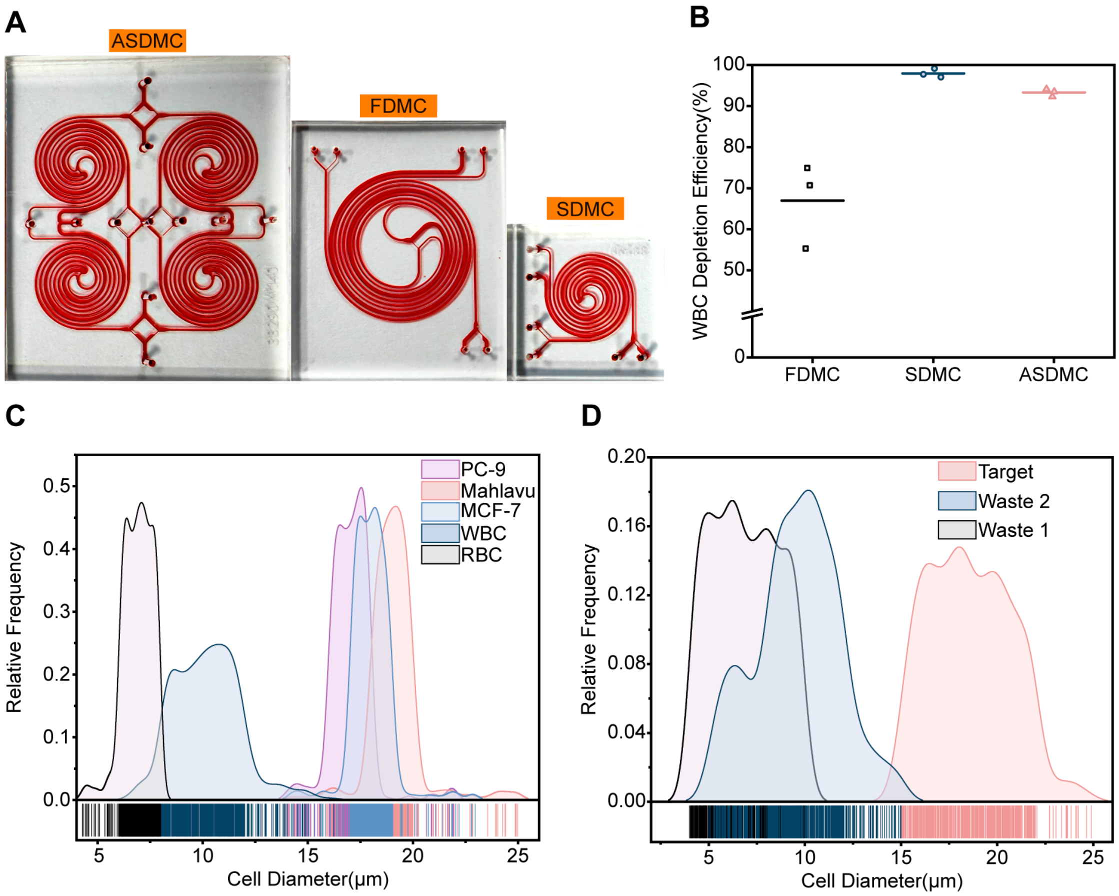

Figure S7.

(A) Comparison of FDMC, SDMC, and ASDMC designs. (B) WBC depletion efficiencies of FDMC, SDMC, and ASDMC. Data are mean ± SD from 3 independent biological replicates per group. (C-D) Size distributions of tumor cells and blood cells (WBCs and RBCs) (n = 500 per cell type) (C) and size distributions of cells collected from different ASDMC outlets regardless of cell type (n = 500 cells per group) (D).

Supplementary Bibliography

(1) Diedrichs, D. R. Archimedean, Logarithmic and Euler Spirals − Intriguing and Ubiquitous Patterns in Nature. *Math. Gaz.* **2019**, *103* (556), 52–64. https://doi.org/10.1017/mag.2019.7.

(2) Di Carlo, D. Inertial Microfluidics. *Lab. Chip* **2009**, *9* (21), 3038–3046. https://doi.org/10.1039/b912547g.

(3) Al-Halhouli, A.; Al-Faqheri, W.; Alhamarneh, B.; Hecht, L.; Dietzel, A. Spiral Microchannels with Trapezoidal Cross Section Fabricated by Femtosecond Laser Ablation in Glass for the Inertial Separation of Microparticles. *Micromachines* **2018**, *9* (4), 171. https://doi.org/10.3390/mi9040171.

(4) Martel, J. M.; Toner, M. Inertial Focusing in Microfluidics. *Annu. Rev. Biomed. Eng.* **2014**, *16* (Volume 16, 2014), 371–396. https://doi.org/10.1146/annurev-bioeng-121813-120704.

(5) Ying, Y.; Lin, Y. Inertial Focusing and Separation of Particles in Similar Curved Channels. *Sci. Rep.* **2019**, *9* (1), 16575. https://doi.org/10.1038/s41598-019-52983-z.

(6) Kye, H. G.; Park, B. S.; Lee, J. M.; Song, M. G.; Song, H. G.; Ahrberg, C. D.; Chung, B. G. Dual-Neodymium Magnet-Based Microfluidic Separation Device. *Sci. Rep.* **2019**, *9* (1), 9502. https://doi.org/10.1038/s41598-019-45929-y.

(7) Shiriny, A.; Bayareh, M. Inertial Focusing of CTCs in a Novel Spiral Microchannel. *Chem. Eng. Sci.* **2021**, *229*, 116102. https://doi.org/10.1016/j.ces.2020.116102.

(8) Di Carlo, D.; Edd, J. F.; Irimia, D.; Tompkins, R. G.; Toner, M. Equilibrium Separation and Filtration of Particles Using Differential Inertial Focusing. *Anal. Chem.* **2008**, *80* (6), 2204–2211. https://doi.org/10.1021/ac702283m.

(9) Zhou, J.; Papautsky, I. Fundamentals of Inertial Focusing in Microchannels. *Lab. Chip* **2013**, *13* (6), 1121–1132. https://doi.org/10.1039/C2LC41248A.

(10) Bhagat, A. A. S.; Kuntaegowdanahalli, S. S.; Papautsky, I. Continuous Particle Separation in Spiral Microchannels Using Dean Flows and Differential Migration. *Lab. Chip* **2008**, *8* (11), 1906–1914. https://doi.org/10.1039/B807107A.
